# Prevalence of Vitamin D Deficiency in Ataxia-Telangiectasia: A Systematic Review and Single Arm Meta-Analysis

**DOI:** 10.1007/s12311-026-02036-9

**Published:** 2026-06-15

**Authors:** Muhammad Junaid Iqbal, Muteeba Azhar, Hammad Javaid, Amina Hassan, Ayesha Masood, Michele Menotta

**Affiliations:** 1 Department of Biomolecular Sciences, University of Urbino “Carlo Bo”, Via Saffi 2, Urbino (PU), 61029 Italy; 2 School of Biochemistry and Biotechnology, University of the Punjab, Lahore, 54000 Pakistan; 3 King Edward Medical University, Lahore, 54000 Pakistan; 4 Department of Medicine, Dow University of Health Sciences, 74200 Karachi, Pakistan; 5 Kinnaird College for Women, Lahore, 54000 Pakistan

**Keywords:** Ataxia-telangiectasia, 25-hydroxyvitamin D, Vitamin D deficiency, Vitamin D insufficiency

## Abstract

Ataxia-telangiectasia (A-T) is a multisystem disorder in which nutritional and endocrine abnormalities are frequently reported. Vitamin D deficiency has been described in A-T cohorts, but its pooled prevalence within published A-T cohorts has not been quantified. We conducted a systematic review and single-arm meta-analysis to estimate the prevalence of vitamin D deficiency in A-T. We systematically identified observational studies reporting serum 25-hydroxyvitamin D 25(OH)D in individuals with A-T. The primary outcome was vitamin D deficiency prevalence using the study-defined threshold of 25(OH)D < 20 ng/mL. Secondary outcomes included severe deficiency (< 10 ng/mL), insufficiency, adequacy, and pooled mean 25(OH)D where available. Random-effects single-arm meta-analyses were performed. Study quality was assessed using the Newcastle–Ottawa Scale. Four studies were included. The pooled prevalence of vitamin D deficiency (< 20 ng/mL) was 50.8% (95% CI 39.4% to 62.2%), with I² = 0%. The pooled prevalence of severe deficiency (< 10 ng/mL) was 26.8% (95% CI 10.4% to 43.1%), with I² = 23%. The pooled prevalence of insufficiency was 21.9% (95% CI 7.6% to 36.1%), with I² = 0%, and adequacy was 38.1% (95% CI 19.5% to 56.8%), with I² = 55.1%. The pooled mean 25(OH)D was 19.65 ng/mL (95% CI 16.26 to 23.04), with I² = 30.7%. Vitamin D deficiency is common in published A-T cohorts, with approximately half meeting a < 20 ng/mL threshold. These findings support routine clinical attention to 25(OH)D in A-T, while highlighting the need for standardized, outcome-focused prospective studies and interventional trials.

**Graphical Abstract:**

**Figure:**
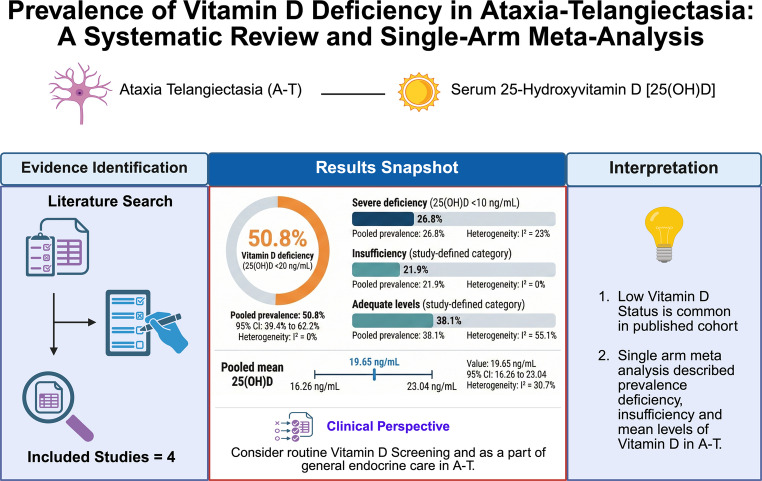

**Supplementary Information:**

The online version contains supplementary material available at 10.1007/s12311-026-02036-9.

## Introduction

Ataxia-telangiectasia (A-T) is a rare autosomal recessive neurogenetic disorder caused by biallelic pathogenic variants in the ATM gene [1]. Deficiency of the ATM protein leads to genomic instability, chronic oxidative stress, mitochondrial dysfunction and neuroinflammation that collectively contribute to Purkinje cell loss and cerebellar atrophy [2]. Ataxia telangiectasia manifests during early childhood as truncal instability while sitting and walking. Neurological symptoms like axonal neuropathy, extrapyramidal indications, oculomotor apraxia and cognitive impairment are its key characteristics. In addition, affected individuals may have raised levels of blood alpha fetoprotein (AFP) levels, immunological and endocrine problems and an increased risk of cancer [3]. An estimated 1 in 40,000 and 1 in 100,000 live births worldwide are thought to have A-T [4]. A-T has an equal impact on males and females. A-T is the second most prevalent childhood-onset cerebellar neurodegenerative condition after Friedreich’s ataxia [3]. Despite detailed molecular characterization, there is currently no curative therapy and management remains largely supportive. Therefore, identifying modifiable metabolic or nutritional factors that may influence disease progression is of increasing interest.

Vitamin D deficiency is particularly concerning, given its established roles in bone metabolism, immune regulation and neuroprotection. In a cross-sectional controlled study, Cruz et al. (2019) evaluated serum 25-hydroxyvitamin D [25(OH)D] levels in patients with Common Variable Immunodeficiency (CVID) and A–T. They found vitamin D deficiency in A–T and CVID patients. Moreover, vitamin D levels were inversely correlated with body mass index in the A–T group, suggesting that metabolic factors may influence deficiency beyond malnutrition alone [5]. Similarly, Prayle et al. (2017) reported vitamin D deficiency in A–T cases, noting that low levels were unrelated to weight Z-score, thereby implicating reduced sunlight exposure and limited mobility as contributing factors [6]. These findings reinforce earlier work by Nissenkorn et al. (2016), who observed that over half of their A–T cohort exhibited vitamin D deficiency. Collectively, these studies demonstrate that vitamin D deficiency is common among individuals with A–T, though its clinical implications for neurological severity or disease progression remain unclear [7].

At the mechanistic level, vitamin D functions as a neurosteroid hormone, exerting antioxidant, anti-inflammatory and immunomodulatory effects throughout the central nervous system [8–10]. Vitamin D receptors and the 1-α-hydroxylase enzyme are abundantly expressed in the cerebellum, hippocampus and cortex [11]. Experimental studies indicate that vitamin D promotes neuronal survival by up-regulating neurotrophins such as NGF and BDNF, maintaining intracellular calcium balance and suppressing pro-inflammatory cytokines through NF-κB inhibition [12]. Observational studies in other neurodegenerative disorders including multiple sclerosis, Parkinson’s disease, amyotrophic lateral sclerosis and traumatic brain injury have shown association with lower serum vitamin D levels [13]. Despite all these advances, no comprehensive systematic review or meta-analysis has examined the prevalence of serum vitamin D levels in ataxia telangiectasia. Therefore, this systematic review and single-arm meta-analysis aimed to estimate pooled mean serum 25(OH)D levels and the prevalence of vitamin D deficiency, severe deficiency, insufficiency, and adequacy among individuals with A-T.

## Materials and Methods

### Protocol Registration

The PRISMA 2020 guidelines have been followed for this systematic review and meta-analysis. The methodology has already been registered in the database of PROSPERO with the registration number of CRD420251166005 [14, 15]. PRISMA checklist is given as Supplementary File 1.

### Search Strategy

A comprehensive literature search was conducted in the PubMed/MEDLINE, Embase, Scopus and Web of Science for all relevant articles published up to October 2025. The search strategy was independently outlined according to the PRISMA standards by two reviewers. The search strategy included several MeSH terms and keywords related to A-T and Vitamin D such as “ataxia-telangiectasia,” “Vitamin D,” “Vitamin d deficiency,” combined using Boolean operators (AND/OR). Reference lists of included studies and relevant reviews were also screened to identify additional eligible articles. All retrieved records were imported into EndNote X20 for duplicate removal and screening.

### Eligibility Criteria

All retrieved records were imported into EndNote X20 for duplicate removal and screening. Titles and abstracts of retrieved records were reviewed independently by two independent reviewers to assess their eligibility. Full-text manuscript of studies that met the preliminary criteria were obtained and evaluated based on the defined inclusion and exclusion criteria that were already submitted in PROSPERO. Any disagreement between the reviewers was resolved through discussion or when necessary, by consultation with a third reviewer.

The inclusion criteria were formulated based on PICOS (Population, Interventions, Comparison, Outcomes, Study Design). Studies were included if they met the following criteria: (1) enrolled participants of any age with a confirmed diagnosis of ataxia-telangiectasia (A-T) based on clinical features, genetic testing or established diagnostic criteria; (2) reported serum Vitamin D levels (e.g., 25-hydroxyvitamin D) using standardized laboratory assays; (3) evaluated the association between Vitamin D status and A-T, including assessments of deficiency, insufficiency, sufficiency or mean Vitamin D concentrations; (4) adopted an eligible study design, including randomized controlled trials, cohort studies, case-control studies or cross-sectional studies; and (5) were published as full-text, peer-reviewed articles in English or with an accessible English translation.

Studies were excluded if they met any of the following conditions: (1) involved participants without a confirmed diagnosis of A-T, including healthy controls only or patients with other neurodegenerative or immunodeficiency disorders; (2) reported Vitamin D measurements without linking them to A-T status or disease-related outcomes; (3) did not provide extractable or usable numerical data on serum Vitamin D levels; (4) focused on laboratory, preclinical or animal models without human subjects; (5) were designed as case reports, case series with fewer than 10 participants, narrative reviews, systematic reviews, editorials, letters, conference abstracts or studies lacking sufficient data for extraction; and (6) were not available as full-text, peer-reviewed articles in English or lacked an English translation.

### Data Extraction and Outcome Measures

Relevant information including study characteristics, patient demographics, intervention details, author identification and outcomes of this meta-analysis including mean vitamin D levels, vitamin D deficiency (< 20 ng/ml), vitamin D severe deficiency (< 10 ng/ml), vitamin D insufficiency and vitamin D adequate levels. For harmonization, vitamin D deficiency was defined as 25(OH)D < 20 ng/mL (< 50 nmol/L), severe deficiency as < 10 ng/mL (< 25 nmol/L), insufficiency as 21–29 ng/mL (52.5–72.5 nmol/L), and adequacy as ≥ 30 ng/mL (≥ 75 nmol/L). These categories were based on commonly used clinical thresholds and the definitions reported in the included studies. Because international definitions vary, pooled estimates were interpreted descriptively rather than as universal diagnostic categories. All the relevant outcomes were extracted and recorded in a standardized form in Excel sheets by two authors. Any disagreements or inconsistencies were resolved through mutual discussion with a third reviewer.

### Risk of Bias Assessment

Newcastle-Ottawa (NOS) assessment tool for cohort studies, and NOS assessment tool adapted for cross-sectional studies were employed to systematically appraise the eligible studies [16, 17]. The NOS evaluates cohort and cross-sectional studies across three domains, selection of participants (maximum 4 stars), comparability of patient groups (maximum 2 stars), and ascertainment of outcomes (maximum 3 stars). Studies with higher scores were considered to have a low risk of bias. Quality assessment was performed independently by reviewers, and discrepancies were resolved through discussion.

### Outcomes and Statistical Analysis

All statistical analyses were performed using R software (version 4.3.2) with the “metaprop” and “metamean” packages. Given the proportional meta-analysis design, effect sizes were synthesized by pooling data of continuous outcomes (mean vitamin D levels) were analyzed using inverse-variance random-effects models and results were presented as pooled raw mean (MRAW) with corresponding 95% confidence intervals (CIs) and dichotomous outcomes were pooled using proportions with 95% confidence intervals (CIs). A random effect model was used for this study. Statistical heterogeneity was evaluated using X^2^ and I² statistics: I² ≤ 40% was interpreted as low, 41–60% as moderate and > 60% as substantial heterogeneity respectively.

## Results

### Study Selection

**Fig. 1:**
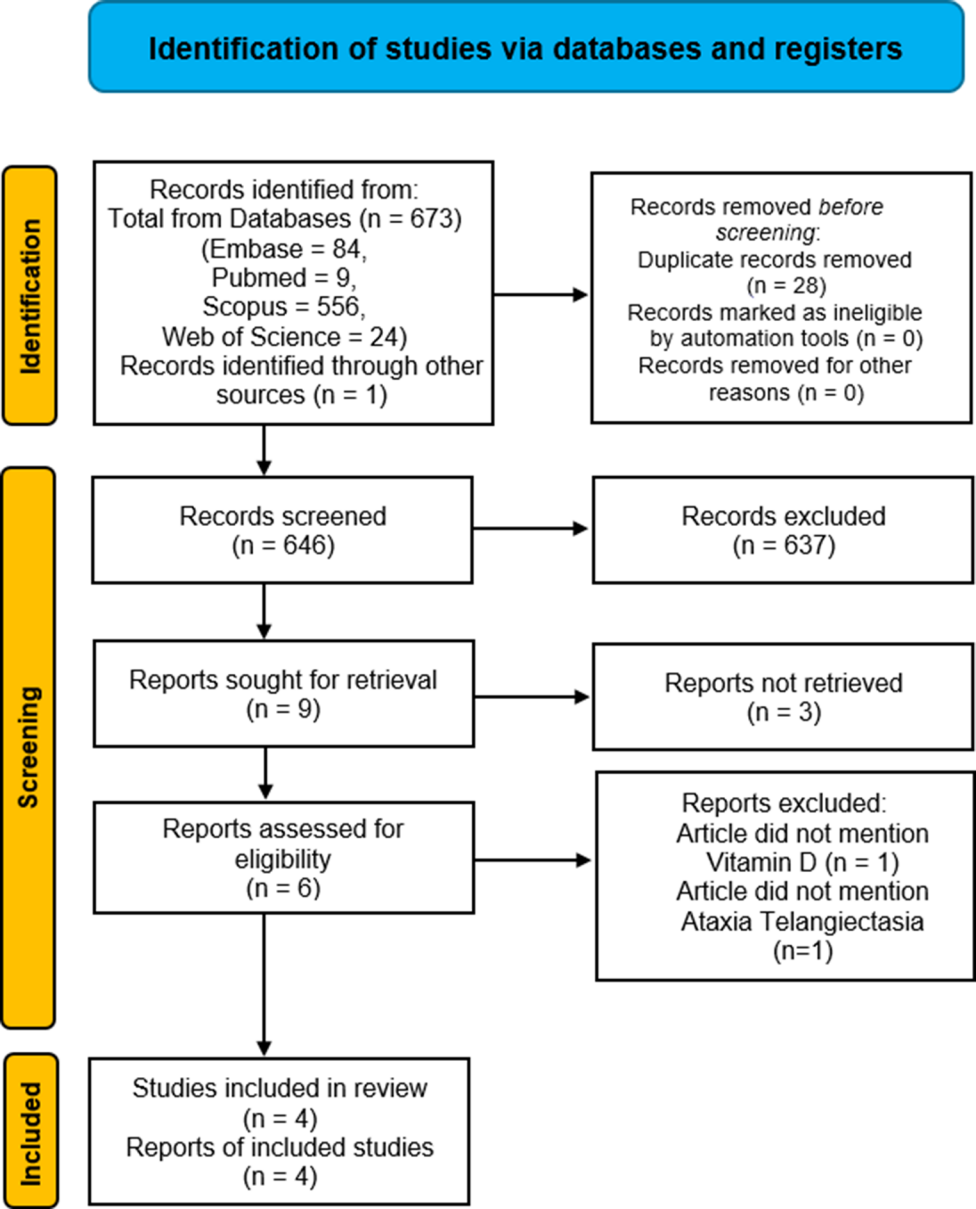
PRISMA flow diagram illustrating the selection process of studies included in the systematic review and meta-analysis of vitamin D status in A-T (made with Microsoft Word)

The PRISMA flow diagram depicts the search strategy and results [18]. During the first search, the database search yielded a total of 673 records (84 from Embase, 9 from PubMed, 556 from Scopus and 24 from Web of Science). One potentially relevant article was not identified during the initial database search, while it is identified though other source. 28 duplicate studies were removed and 646 unique studies considered for screening. It was screened by title and abstract and 637 were excluded due to irrelevance to the study, 9 articles were retrieved after the primary screening, 3 were excluded due to the unavailability of the full text. During secondary screening, one article was excluded because it did not mention vitamin D and another was excluded because it did not mention A-T. After these exclusions, 4 studies met the eligibility criteria and were included in the final meta-analysis. Figure 1.

### Study Characteristics

The clinical data of 99 A-T patients was pooled across the four included cohort studies. The ages of the participants ranged from 2 to 56 years. The biological sex of 49 patients was reported in the eligible studies in the following proportion: 26 (53.1%) males and 23 (46.9%) females. The study characteristics are outlined in Table 1.

**Table 1:** Study Characteristics of Eligible Studies

Year of publication	Country	Study design	Number of A-T patients	Age	Methodology used to assess Vitamin D levels	Criteria for diagnosis	Reference
2014	Qatar & Italy	Cross sectional (observational cohort)	13	Mean: 7.7 ± 3.5 years Range: 3-14.5 years	Not mentioned	Deficiency: serum 25-hydroxyvitamin D level < 20 ng/ml	[19]
2015	Germany	Cross-sectional cohort study	24	Mean 13.04 ± 6.79 years	Chemiluminescence immunoassay using the IMMULITE 1000 Immunoassay System	World Health Organization (WHO) recommendations	[20]
2016	Israel	Retrospective cohort study	23	Range: 2-26.2 years	Not mentioned	Deficient: <15ng/mL Insufficient: 15–20 ng/mL Sufficient: > 20 ng/mL	[7]
2019	Brazil	Cross-sectional and controlled	9	Range: 8–56 years (CVID and A-T samples) Median: 26 years	Electrochemiluminescence	Deficient: <20ug/dL Insufficient: 21–29ug/dL Sufficient: ≥ 30 ug/dL	[5]

The four included studies (2014–2019) were conducted in Brazil, Israel, Qatar, Germany and Italy with sample sizes ranging from 9 to 24 A-T patients, some including matched controls. Participants were of both genders within the age of 2 to 56 years. Across studies, BMI values reflected generally poor nutritional status with mean/median BMI ranging from 15.1 to 17.3 kg/m² and up to 44% of patients falling below the 3rd percentile. Vitamin D assessment methods varied, including electrochemiluminescence and chemiluminescence immunoassay, while two studies did not specify the technique. A-T diagnosis in all studies was based on established clinical or WHO-referenced criteria.

### Quality Assessment of Eligible Studies

We assessed methodological quality using the Newcastle Ottawa Scale (NOS). Across the four included studies, total NOS scores ranged from 6 to 8 stars out of 10, indicating moderate to good quality overall, with important limitations in selection and confounding control as shown in Table 2. The detailed assessments in visual forms are presented in Supplementary Figures S1 as (A) ‘Traffic Light Plot’ and (B) ‘Summary Plot’ respectively.

**Table 2:** Quality Assessment of Eligible Studies using Newcastle-Ottawa Scale

Study	Selection (Representativeness, sample size, non-respondents, exposure)	Comparability (Confounders controlled)	Outcome (Outcome assessment, Statistical test)	Total stars
[19]	★★/5	★★/2	★★/3	6★/10
[7]	★★★★/5	★★/2	★★/3	8★/10
[5]	★★★/5	★★/2	★★★/3	8★/10
[20]	★★★/5	★★/2	★★★/3	8★/10

### Vitamin D Status

Extracted vitamin D outcomes are summarized in Table 3. Meta-analytic results are presented below for pooled mean 25(OH)D and prevalence categories.

**Table 3:** Vitamin D extracted outcomes for single arm meta-analysis

Study	Mean 25(OH)D ng/mL (SD)	Deficiency < 20 ng/ml	Severe deficiency < 10 ng/ml	Insufficient	Adequate
Cruz 2019	24.80 (10.50)	4/9 (44.4%)	NR	2/9 (22.2%)	3/9 (33.3%)
Nissenkorn 2016	18.70 (10.90)	12/23(52.2%)	NR	5/23 (21.7%)	6/23 (26.1%)
Ehlayel 2014	NR	8/13 (61.5%)	5/13 (38.5%)	NR	NR
Pommerening 2015	18.08 (10.09)	11/24 (45.8%)	5/24 (20.8%)	NR	13/24 (54.2%)

#### Mean Vitamin D Levels

**Fig. 2:**
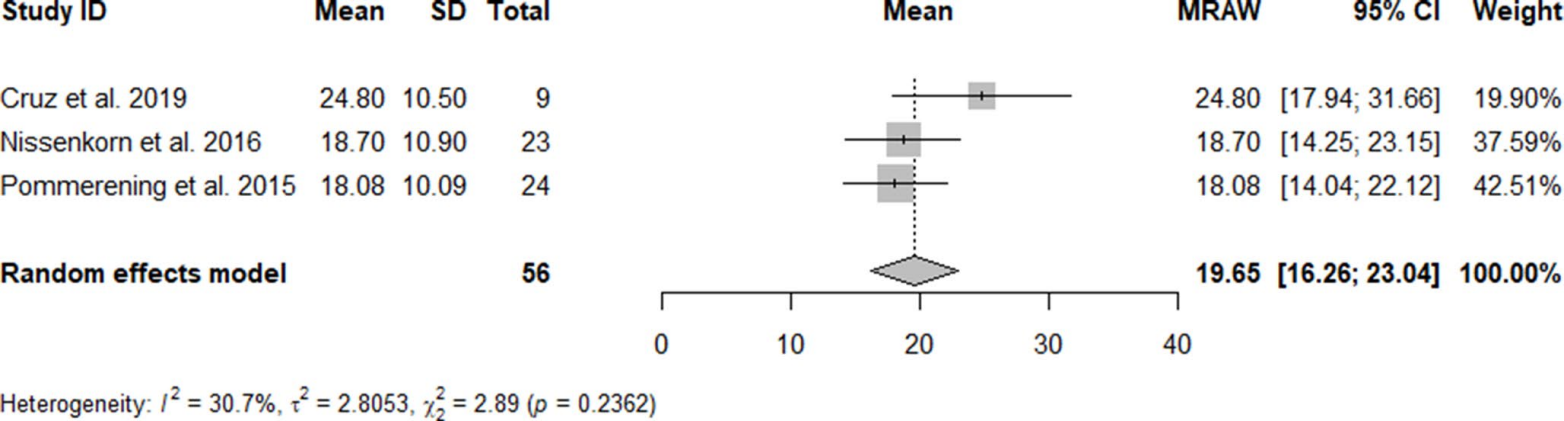
Forest plot showing pooled mean serum 25-hydroxyvitamin D levels in patients with ataxia telangiectasia from three studies. (Made with R software, version 4.3.2)

Three included studies reported mean vitamin D levels in 56 patients with A-T. A pooled analysis revealed a mean level of Vitamin D of 19.65ng/ml (95% CI 16.26ng/ml to 23.04ng/ml) in patients with A-T. I^2^ = 30.7% showed that there was low to moderate interstudy heterogeneity as shown in Fig. 2.

#### Vitamin D Deficiency (< 20 ng/ml)

**Fig. 3:**
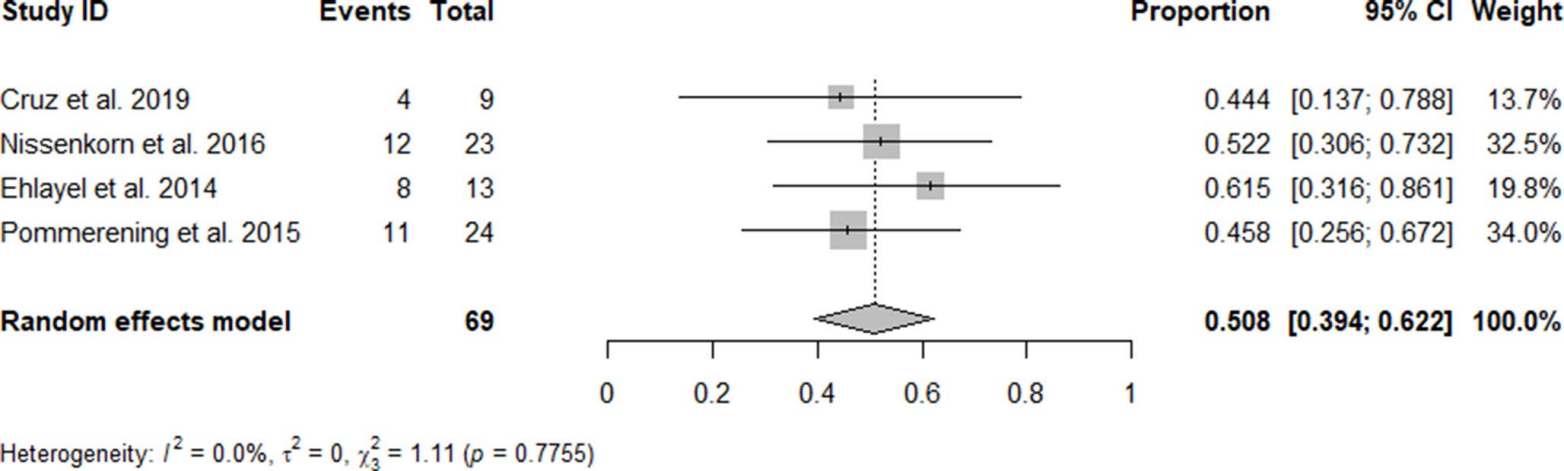
Forest plot of the pooled prevalence of vitamin D deficiency (<20 ng/mL) among patients with ataxia telangiectasia across four studies. (Made with R software, version 4.3.2)

All the included studies reported vitamin D deficiency (< 20 ng/ml) in 69 patients suffering from ataxia telangiectasia. A pooled analysis showed vitamin D Deficiency (< 20 ng/ml) in 50.8% A-T patients (95% CI 39.4% to 62.2%). I^2^ = 0% showed that there was low interstudy heterogeneity as shown in Fig. 3.

#### Vitamin D Severe Deficiency (< 10 ng/ml)

**Fig. 4:**
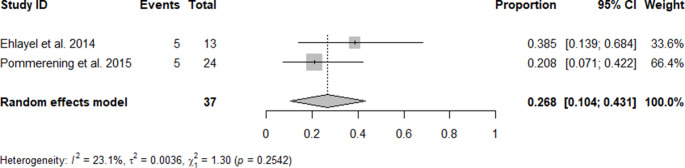
Forest plot showing pooled prevalence of severe vitamin D deficiency (< 10 ng/mL) in patients with ataxia telangiectasia from two studies. (Made with R software, version 4.3.2)

Two included studies reported vitamin D deficiency (< 10 ng/ml) in 37 patients suffering from ataxia telangiectasia. A pooled analysis showed vitamin D Deficiency (< 10 ng/ml) in 26.8% A-T patients (95% CI 10.4% to 43.1%). I^2^ = 23% showed that there was low interstudy heterogeneity as shown in Fig. 4.

#### Vitamin D Insufficiency

**Fig. 5:**
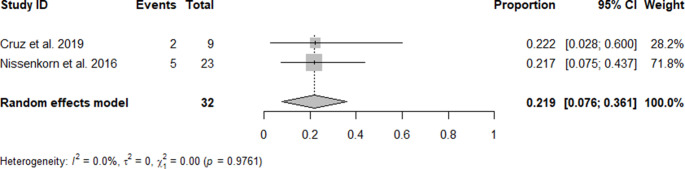
Forest plot showing pooled prevalence of vitamin D insufficiency in patients with ataxia telangiectasia from two studies. (Made with R software, version 4.3.2)

Two included studies reported vitamin D insufficiency in 32 patients suffering from ataxia telangiectasia. A pooled analysis showed the prevalence of vitamin D insufficiency was 21.9% (95% CI 7.6% to 36.1%) in patients with A-T. I^2^ = 0% showed that there was no interstudy heterogeneity as shown in Fig. 5.

#### Vitamin D Adequate Levels

**Fig. 6:**
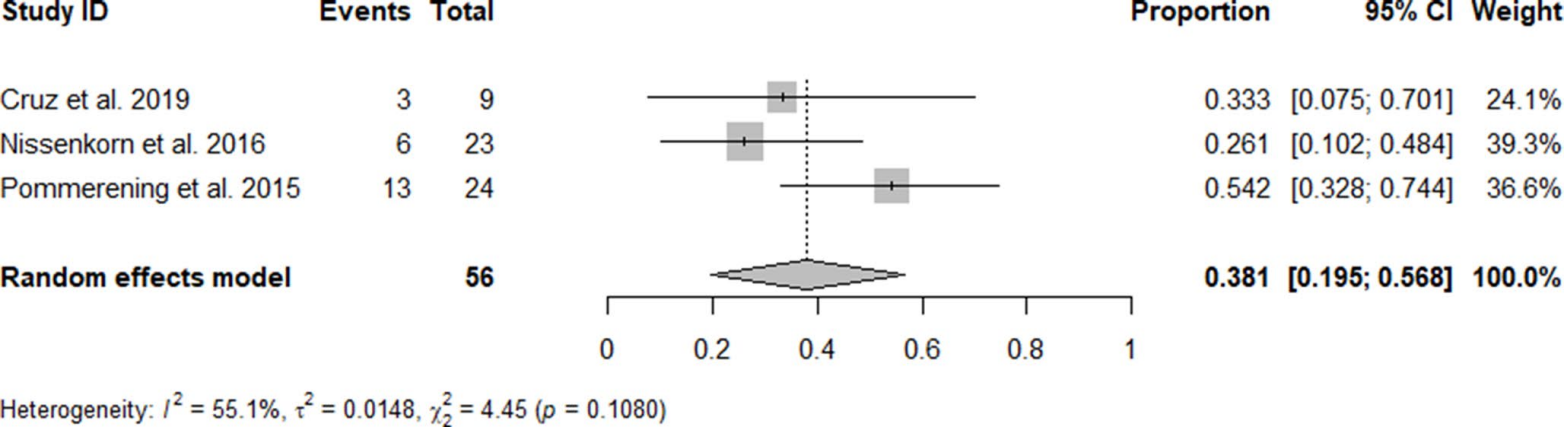
Forest plot shows pooled prevalence of adequate vitamin D levels in patients with ataxia telangiectasia from three studies. (Made with R software, version 4.3.2)

Three included studies reported the prevalence of vitamin D adequate levels in 56 patients with A-T. A pooled analysis revealed the prevalence of vitamin D adequate levels was 38.1% (95% CI 19.5% to 56.8%) in patients with A-T. I^2^ = 55.1% showed that there was moderate inter study heterogeneity as shown in Fig. 6.

## Discussion

In this systematic review and single-arm meta-analysis of observational A-T cohorts, low vitamin D status was frequent when defined by serum 25-hydroxyvitamin D (25(OH)D) < 20 ng/mL. The pooled prevalence of deficiency was 50.8% (95% CI 39.4% to 62.2%), with I² = 0%, and the pooled mean 25(OH)D concentration was 19.65 ng/mL (95% CI 16.26 to 23.04) with I² = 30.7%. Severe deficiency (< 10 ng/mL) was also observed, with a pooled prevalence of 26.8% (95% CI 10.4% to 43.1%) and I² = 23%. In contrast, the pooled prevalence of vitamin D adequacy showed greater between study variability at 38.1% (95% CI 19.5% to 56.8%), I² = 55.1%, while insufficiency prevalence was 21.9% (95% CI 7.6% to 36.1%), I² = 0%. These pooled estimates support a descriptive conclusion, low vitamin D status is frequent in published A-T cohorts, and the average vitamin D level lies near widely used clinical deficiency thresholds. The inference should remain narrowly bounded. A single arm prevalence meta-analysis quantifies how common deficiency is within A-T samples, it does not estimate the excess risk relative to matched controls, and it does not imply causality. PRISMA 2020 emphasizes transparent interpretation of what a synthesis can and cannot show, particularly when the evidence base is small and context dependent [21]. Prevalence meta-analyses are also sensitive to setting and measurement context, and summary prevalence is most informative when between-study context is sufficiently comparable [22–24].

A few studies measured vitamin D in the broader context of endocrine function, growth, and body composition [7, 19, 20]. In the national A-T cohort described by Nissenkorn and colleagues, vitamin D deficiency was explicitly reported as frequent, and the authors recommended that vitamin D levels should be followed and supplementation given if needed [7]. This supports the clinical relevance of vitamin D assessment within broader endocrine and nutritional care in A-T, but it should not be interpreted as evidence that deficiency is specific to A-T. Adiposity-related mechanisms may contribute in some patients, particularly because vitamin D is fat soluble and lower circulating 25(OH)D concentrations are consistently observed in obesity. Mechanistic work suggests decreased bioavailability of vitamin D from cutaneous and dietary sources due to deposition in adipose compartments [25]. Some studies further discuss distribution and sequestration as plausible contributors to lower circulating levels in obesity [26]. Where A-T cohorts report associations between lower vitamin D and higher body fat percentage or BMI, this is consistent with established obesity and vitamin D epidemiology rather than a disease-specific mechanism, and should be interpreted accordingly [20, 25].

Chronic neurologic and disability associated conditions often report high rates of vitamin D deficiency, possibly due to reduced outdoor exposure, nutritional compromise, and comorbidity burden. A recent meta-analysis in children with cerebral palsy reported high prevalence of vitamin D deficiency, although the clinical contexts differ substantially from A-T [27]. In neuromuscular disorders such as Duchenne muscular dystrophy, vitamin D insufficiency and deficiency are frequently discussed within broader skeletal health frameworks, often in the context of reduced mobility and corticosteroid exposure [28]. These comparators do not prove that A-T causes deficiency, but they provide a realistic clinical lens in chronic pediatric neuro-disability and neuromuscular disease that vitamin D deficiency is frequently encountered, and prevalence in the range observed here is biologically and clinically plausible. Within A-T-specific studies, vitamin D status has been reported as part of endocrine and nutritional profiling rather than as the primary research question [5, 7, 19, 20]. The Nissenkorn national cohort provides the clearest A-T-specific observational evidence because it explicitly quantified deficiency frequency within endocrine abnormalities in A-T [7]. A separate A-T and CVID comparison study also supports that low vitamin D is a recurring observation in immunodeficiency clinic populations, though thresholds, supplementation, and selection frames differ [5].

Statistical heterogeneity was minimal for deficiency prevalence (< 20 ng/mL; I² = 0%) and for insufficiency prevalence (I² = 0%), modest for severe deficiency (I² = 23%), and moderate for adequate vitamin D levels (I² = 55.1%). In prevalence meta-analysis, I² should be interpreted cautiously, since it is influenced by the number of studies, the magnitude of the pooled prevalence, and between-context differences that are often inevitable when pooling across settings. Low I² does not guarantee clinical homogeneity, particularly with few studies, and reviewers should emphasize contextual comparability and expected ranges rather than relying on arbitrary thresholds [23]. Several plausible drivers of between-study variability are particularly relevant for vitamin D. First, assay variability is non-trivial. Measured 25(OH)D concentrations vary by assay type and laboratory, and standardization efforts exist precisely because lack of comparability can distort deficiency classification around clinical thresholds [29, 30]. In small rare-disease cohorts, even modest assay bias can change how many patients fall below 20 ng/ml. Second, season and latitude are major determinants of population vitamin D distributions and can shift deficiency prevalence substantially, affecting cross-country comparability [31, 32]. Third, patient-level determinants, including supplementation, diet, mobility, and adiposity, may differ by center and are inconsistently recorded in observational cohorts. The obesity and vitamin D relationship is robustly documented, and differences in body composition across cohorts could contribute to differences in mean 25(OH)D and the proportion below thresholds [25, 26].

Given the pooled prevalence of deficiency and severe deficiency, clinicians caring for individuals with A-T should consider periodic assessment of serum 25(OH)D as part of broader endocrine, nutritional, and skeletal health surveillance, particularly in patients with limited mobility, recurrent infections, growth concerns, or other risk factors. However, the present meta-analysis does not test whether correcting deficiency improves A-T-specific clinical outcomes, and prevalence of deficiency should not be conflated with evidence of therapeutic benefit. Evidence outside A-T suggests that supplementation effects depend on baseline deficiency and dosing regimen for some outcomes. For example, an individual participant data meta-analysis reported reduced risk of acute respiratory tract infection with vitamin D supplementation overall, with stronger effects among those with low baseline levels and with daily or weekly dosing rather than bolus schedules [33]. This supports biological plausibility for studying supplementation in immunologically vulnerable groups, but it is not direct evidence for benefit in A-T. Therefore, in A-T, supplementation should be framed primarily as correction of deficiency for general health, bone and muscle considerations, and alignment with standard care for deficiency, rather than as a proven disease-modifying intervention.

## Strengths and Limitations

A key strength is that this work consolidates sparse, dispersed evidence in an ultra-rare disease, generating quantitative estimates of deficiency prevalence and mean 25(OH)D levels. The included studies represent different clinical settings and measured vitamin D in cohorts relevant to endocrine and nutritional care in A-T [5, 7, 19, 20]. Use of a structured quality tool (Newcastle-Ottawa Scale) adds transparency regarding internal validity concerns. Several limitations are also there impacting this meta-analysis. First, the number of studies and participants is small, which increases imprecision and makes heterogeneity statistics unstable, particularly for category outcomes such as severe deficiency and adequacy. Second, designs are observational and largely cross-sectional cohorts, precluding causal inference and limiting control for confounding by season, supplementation, mobility, adiposity, and disease severity. Third, assay and laboratory variability in 25(OH)D measurement may bias classification around clinical thresholds, and standardization is not assured across cohorts. Fourth, representativeness is uncertain, since cohorts are drawn from specialty centers and may oversample more severe phenotypes. Fifth, the synthesis was single-arm and did not include harmonized matched-control comparisons; therefore, it cannot determine whether A-T patients have excess risk of vitamin D deficiency compared with healthy children or other chronic pediatric populations. Finally, the synthesis focuses on vitamin D status rather than clinical endpoints such as bone mineral density, fractures, pulmonary outcomes, infections, or neurologic progression, so clinical impact of deficiency in A-T remains unquantified in this evidence base.

## Conclusion

This systematic review and single-arm meta-analysis indicates that low vitamin D status is frequent in published cohorts of individuals with ataxia-telangiectasia. Approximately half of the included A-T patients met the study-defined threshold for vitamin D deficiency (< 20 ng/mL), and the pooled mean 25(OH)D level was close to commonly used deficiency cutoffs. These findings should be interpreted as descriptive evidence of vitamin D status in A-T rather than as proof of an A-T-specific association, causal relationship, or disease-modifying effect. The available evidence is limited by the small number of observational studies, modest sample size, variable measurement context, lack of harmonized control groups, and absence of pooled clinical outcome data. Nevertheless, the results support consideration of 25(OH)D assessment as part of broader endocrine, nutritional, and skeletal surveillance in A-T, particularly because vitamin D deficiency is potentially correctable and clinically relevant to general bone and nutritional health. Future studies should use prospective designs, standardized 25(OH)D assays, season-adjusted sampling, clear reporting of supplementation status, matched comparator groups, and clinically meaningful outcomes such as bone health, growth, infection burden, respiratory morbidity, and functional status. Such evidence is needed before vitamin D management in A-T can move beyond general deficiency correction toward disease-specific clinical recommendations.

## Supplementary Information

Below is the link to the electronic supplementary material.

Supplementary Material 1

Supplementary Material 2

Supplementary Material 3

## Data Availability

No datasets were generated or analysed during the current study.

## Declarations

### Prospero Registration Number

CRD420251166005
