# Supplementary figures and images for: Prevalence of Vitamin D Deficiency in Ataxia-Telangiectasia: A Systematic Review and Single Arm Meta-Analysis
Source: Cerebellum. 2026 Jun 15;25(4):94. doi: 10.1007/s12311-026-02036-9 (PMC13269378; doi:10.1007/s12311-026-02036-9)

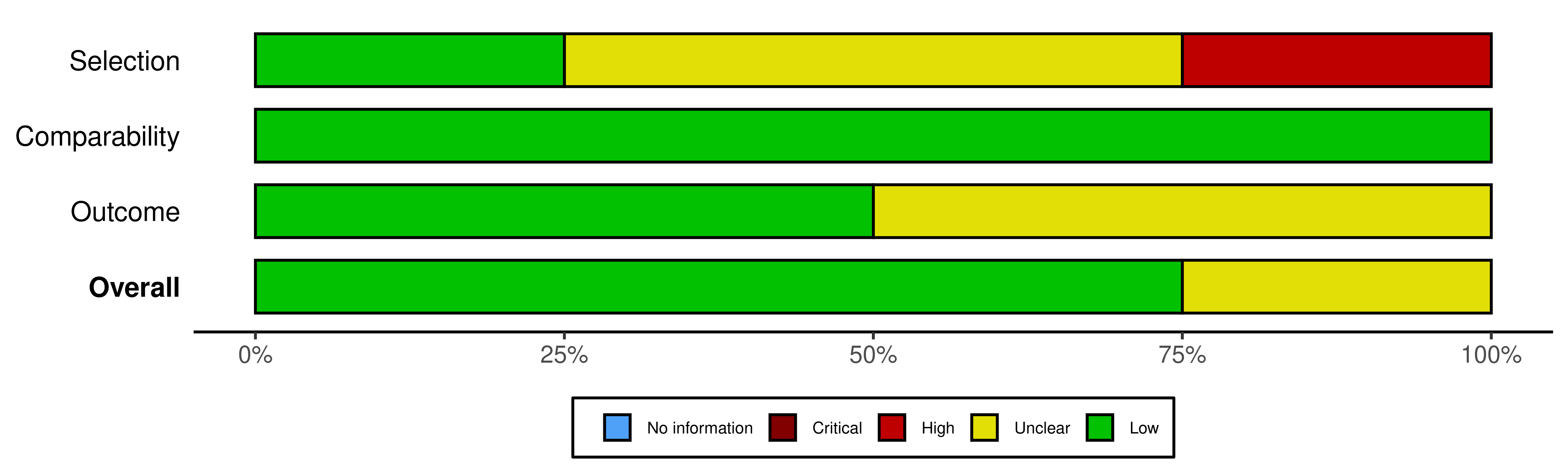

Supplement: Supplementary file 2 — Supplementary Material 2 [file 12311_2026_2036_MOESM2_ESM.png]

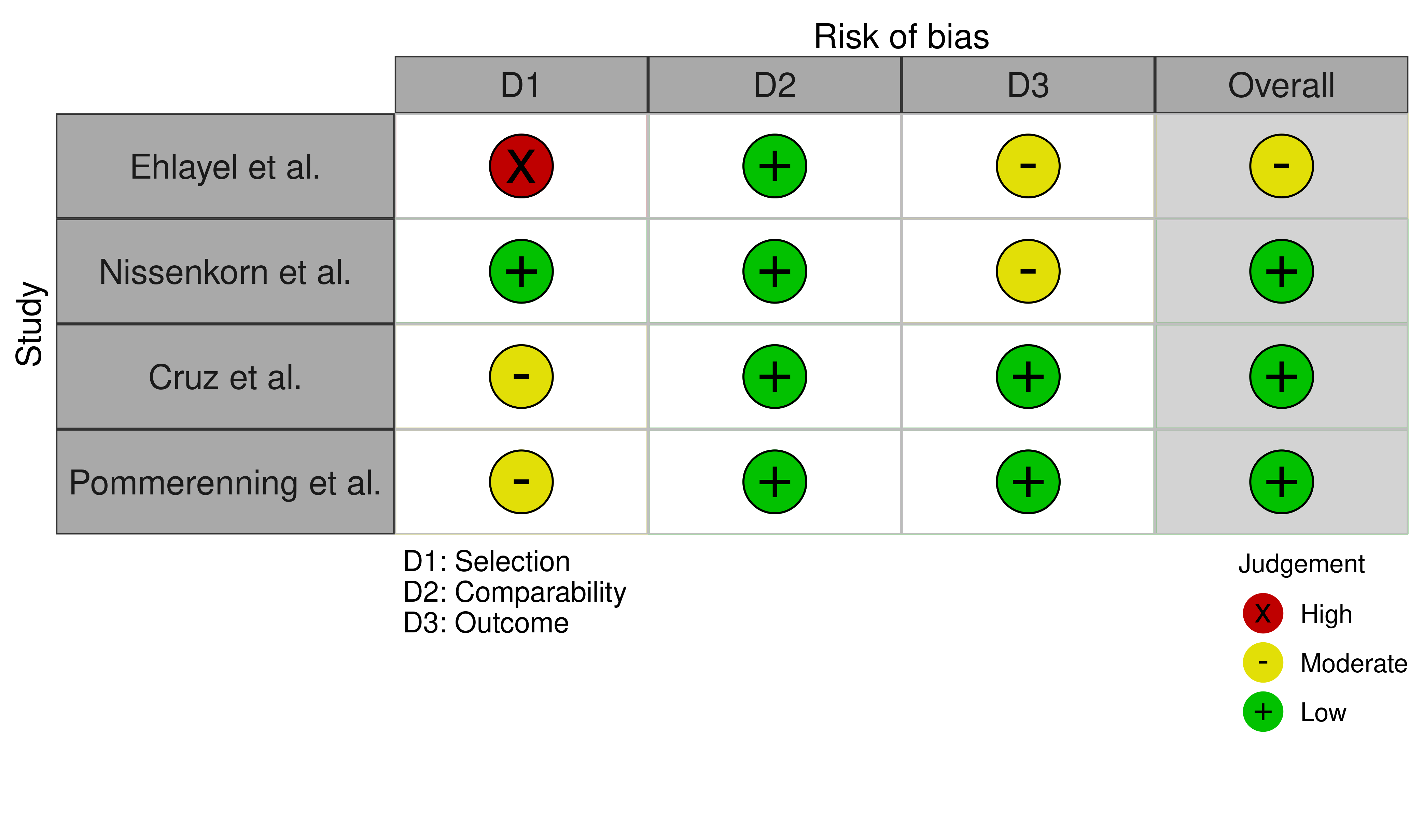

Supplement: Supplementary file 3 — Supplementary Material 3 [file 12311_2026_2036_MOESM3_ESM.png]
